# Safety Planning Interventions for Suicide Prevention in Children and Adolescents: A Systematic Review and Meta-Analysis

**DOI:** 10.1001/jamapediatrics.2025.1012

**Published:** 2025-05-19

**Authors:** Carly Albaum, Samantha H. Irwin, Jessica Muha, Anett Schumacher, Sherinne Clarissa, Yaron Finkelstein, Jeffrey A. Bridge, Daphne J. Korczak

**Affiliations:** 1 Centre for Addiction and Mental Health, Toronto, Ontario, Canada; 2 SickKids Research Institute, Toronto, Ontario, Canada; 3 Temerty Faculty of Medicine, University of Toronto, Toronto, Ontario, Canada; 4 Department of Pediatrics, Hospital for Sick Children, Toronto, Ontario, Canada; 5 Abigail Wexner Research Institute at Nationwide Children’s Hospital, Columbus, Ohio; 6 The Ohio State University College of Medicine, Columbus; 7 Department of Psychiatry, Hospital for Sick Children, Toronto, Ontario, Canada

## Abstract

**Importance:**

Suicide ideation and suicide-related behavior (eg, suicide attempts) are increasingly prevalent among adolescents. Evidence supports safety planning interventions for adults at risk for suicide; the effectiveness for adolescents is unknown.

**Objective:**

To evaluate the effectiveness of safety planning as a standalone intervention for adolescents with suicide ideation and/or suicide-related behavior.

**Data Sources:**

Ovid MEDLINE, OVID PsycINFO, EBSCO CINAHL, and Scopus (Elsevier) from January 1, 2008, to March 26, 2024.

**Study Selection:**

Included were all studies that examined safety planning as a standalone treatment for adolescents and assessed suicide ideation, suicide-related behavior including suicide attempts, and/or re-presentations to health care settings. Included were peer-reviewed studies evaluating intervention effectiveness or efficacy, with or without a control condition. Nonempirical studies, gray literature, and studies not available in English were excluded.

**Data Extraction and Synthesis:**

Two reviewers performed data extraction and quality assessment independently. Meta-analytic random-effects models were used to calculate effect size estimates. Risk of bias was assessed using Joanna Briggs Institute Critical Appraisal tools.

**Main Outcomes and Measures:**

The outcomes planned for extraction were suicide ideation, suicide-related behavior (eg, attempts; planning with intent to act), and re-presentation to health care settings (ie, emergency department visit; inpatient admission) during the follow-up period.

**Results:**

Ten studies including 1002 adolescents (mean [SD] age, 15.0 [0.4] years; 76.0% female) met inclusion criteria; 5 were included (n = 619) in the meta-analysis. There was no significant association between safety planning interventions and suicide ideation (Hedges *g* = 0.11; 95% CI, 0.01-0.21), behavior (Hedges *g* = −0.09; 95% CI, −0.20 to 0.02), attempts (risk ratio [RR], 0.81; 95% CI, 0.24-2.67) or suicide-related re-presentation (RR, 0.99; 95% CI, 0.29-3.35) at follow-up. Risk of bias for the majority of studies was moderate to high.

**Conclusions and Relevance:**

This systematic review and meta-analysis found limited research evaluating safety planning with adolescents. Although available data do not support safety planning as a standalone treatment for reducing suicide-related outcomes among children and adolescents, well-powered studies are needed to address this common intervention for suicide prevention in adolescents.

## Key Points

**Question:**

What is the effectiveness of safety planning as a standalone intervention for suicide prevention in children and adolescents?

**Findings:**

In this systematic review and meta-analysis of the 5 studies that evaluated interventions, safety planning was not associated with reductions in suicide ideation, suicide-related behavior, suicide attempts, or suicide-related re-presentation to health care settings (eg, emergency departments; inpatient units) at follow-up. There was moderate to high risk of bias in the included studies.

**Meaning:**

Current evidence to support safety planning as an effective intervention for children and adolescents at increased risk of suicide is limited.

## Teaser

This systematic review and meta-analysis investigates the effectiveness of safety planning as a standalone intervention for suicide prevention in children and adolescents.

## Introduction

Globally, we have witnessed a surge in suicide ideation (SI), suicide-related behaviors (SRBs; eg, planning with intent to act; self-harm), and suicide attempts (SAs) in adolescents (ie, 10-19 years old).^1,2,3,4^ Health care utilization (ie, emergency department and inpatient encounters) for self-injurious thoughts and behavior (SITB) in adolescents has also increased in recent years,^5,6,7^ with US data indicating a relative increase of 163.2% in pediatric hospitalizations for self-harm or attempted suicide from 2009 to 2019.^8^ Adolescents who present to emergency departments or who are admitted to hospital for SITB are at increased risk for re-presentation to acute care settings for future suicide attempts.^9,10^ Further, health care expenditures related to SI/SRB in adolescents are pronounced, with SI-related emergency department visits for children and adolescents accounting for $785 million in charges over a 5-year period at a single hospital.^11^ In 2019, the estimated total cost of pediatric mental health hospitalizations was $1.37 billion, of which suicide or self-injury accounted for 8.8%.^8^ There is a pressing need for accessible evidence-based interventions to address SITB and reduce re-presentation among adolescents.

Safety planning interventions (SPIs) are brief treatments that aim to prevent or reduce SI and SRB. Safety planning, developed as a component of cognitive therapy for suicidal adults,^12^ is now routinely used as a standalone treatment for adolescents^13^ in a variety of contexts including emergency departments, inpatient psychiatric units, outpatient and trauma treatment centers, and crisis hotlines.^14^ Generally, SPIs incorporate several key components: recognizing warning signs or triggers of crisis, determining internal and external coping strategies, identifying social contacts or settings that can provide distraction during crisis, recording contact information for professionals, agencies, and local emergency treatment facilities, and restricting access to lethal means.^14^ Although it is suggested that SPIs include all components,^14^ the overall fidelity to the SPI model in clinical practice varies across settings and practitioners.^15^ Evidence supports the use of SPI for adults at risk for suicide^16^ and are included in practice guidelines recommended by the National Institute for Health and Care Excellence for mitigating self-harm.^17^

The evidence base for use of SPIs in adolescents is unclear. Few studies have evaluated the effectiveness of SPIs as a standalone intervention for suicide-related outcomes for children and adolescents. A recent meta-analysis^16^ aimed to determine whether SPIs are associated with improvement in SRB and SI across all age groups. Despite this aim, however, the search did not yield any studies that included children and adolescents. This may have been due in part to stringent inclusion criteria, which required studies to have applied a control condition. Given the limited volume of rigorous controlled trials on SPIs for adolescents, it is important to examine findings from a broader range of study designs to describe current knowledge regarding the SPI effectiveness in this age group. A recent scoping review^18^ provided a narrative overview of literature published between 2008 and 2020 that reported on the effectiveness of safety planning for children and young people. It indicated that there is some research supporting the use of SPIs, however, effectiveness was liberally defined as “…having the ability to do more good than harm for the target population in a real-world setting…,”^18^^(p901)^ and estimates of the magnitude of the effect were not provided.^18^ At present, the question of whether SPIs are an evidence-based standalone approach for reducing suicide-related outcomes in children and adolescents has not been clearly answered.

We sought to systematically review and meta-analyze the available research describing treatment effectiveness or efficacy of standalone SPIs for children and adolescents with acute SI or SRB. We focused on research that quantitatively evaluated suicide-related outcomes (ie, SI, SRB, SA) and rates of re-presentation to acute care settings (ie, emergency department, inpatient unit) after receipt of an SPI to inform future research and standards for clinical care of children and adolescents who present with SITB.

## Methods

### Search Strategy

A systematic review of published studies examining SPIs as a standalone treatment for SITB among children and adolescents was conducted in accordance with the Preferred Reporting Items for Systematic Reviews and Meta-analyses (PRISMA) reporting guidelines.^19^ The protocol was prospectively registered with the PROSPERO International Prospective Register of Systematic Reviews (CRD42024527315). A concurrent search of Ovid MEDLINE, OVID PsycINFO, EBSCO CINAHL, and Scopus (Elsevier) was conducted from January 1, 2008, to March 26, 2024. Electronic search strategies were developed in collaboration with an academic health sciences librarian, and peer-reviewed according to the Peer Review of Electronic Search Strategies (PRESS) guidelines.^20^ The search strategy included terms describing sample age (eg, child*, youth, adolescen*, teen), intervention (eg, safety plan*, crisis plan*, coping plan* suicid* intervention, SPI), and suicide- and re-presentation-related outcomes (eg, suicid*, suicid* ideation*, hospitaliz*, self-harm). When possible, terms were modified to align with database-specific index terms. The full search strategy is reported in eTable 1 in Supplement 1. Reference lists of articles that met inclusion criteria were reviewed to identify additional relevant studies not found through database search.

### Selection Criteria and Data Extraction

Based on previous research,^16,18^ studies were examined based on the following inclusion criteria: (1) a brief standalone intervention focused on safety planning for suicide prevention was delivered, (2) the safety plan was the primary element of the intervention, (3) the study sample comprised children and adolescents experiencing SI and/or SRB with a mean age of 19 years or younger, and (4) the study reported on at least 1 validated and interpretable outcome of SI, SRB (eg, intent to act with plan; self-harm), SA, or re-presentation to acute care during the follow-up period. Peer-reviewed studies evaluating intervention effectiveness or efficacy, with or without a control condition, were included. Qualitative and nonempirical studies (eg, case reports, unpublished dissertations or theses, systematic reviews) were excluded. Due to limited capacity within the study for a translator, only studies published in the English language were included in this review.

Titles and abstracts of articles retrieved through database searches were independently screened by 2 reviewers (C.A., S.H.I., A.S., S.C.). All articles that were retained from the title and abstract screening underwent full-text review. Full texts were independently reviewed by 2 authors (C.A., S.H.I.) with interrater agreement of 88%. Disagreements were resolved through discussion. Data were extracted by 2 independent reviewers (C.A., S.H.I.) and checked for consistency using Covidence software.^21^ Disagreements were resolved through discussion, and, when necessary, by a third reviewer (D.K.). Authors of included studies were contacted via email to request outcome data when not reported in the article. Data on race and ethnicity were extracted to capture sample diversity of included studies.

### Quality Appraisal of Included Studies

The Joanna Briggs Institute (JBI) Critical Appraisal Tools were used to determine methodological quality and possibility of bias for included studies. The JBI was used as a quality appraisal tool in other review studies on adolescents suicide prevention.^18^ Reviewers indicated “yes” or “no” or “unclear” to several methodology-related criteria based on study design (eg, quasi-experimental vs randomized controlled trial),^22,23^ and available information. The quality of each study was based on responses to individual criterion vs a global rating or overall score. Studies with less than 25% of items rated as yes were considered high risk of bias, those with 25% to 75% of items rated yes were moderate, and those with more than 75% of items rated yes were low risk of bias. Quality appraisal for included studies was completed by 2 reviewers (C.A., S.H.I.), with strong agreement across raters (Fleiss κ = 0.96).

### Statistical Analyses

Effect sizes were calculated and analyzed using R statistical software (R Project for Statistical Computing) and the metafor package.^24,25^ For categorical outcomes (ie, SA, re-presentation), effect sizes were computed as risk ratios (RRs) from 2 × 2 tables for meta-analyses.^26^ For continuous outcomes (ie, SI, SRB), Hedges *g* was used to standardize results across all studies. Effect sizes were primarily generated based on applying the Hedges *g* correction factor to a calculated standard mean difference.^27^ When necessary, reported odds ratios or χ^2^ statistics were converted to Hedges *g*.^28^ Hedges *g* was chosen over Cohen *d* to mitigate the effect of overestimation in studies with small sample sizes *(n < *50).^29^ Values of 0.2, 0.5, and 0.8 were considered small, moderate, and large in magnitude, respectively.^30^ Effect size estimates were based on a random-effects model; Hartung-Knapp adjustments were applied to calculate the CIs.^31^ Study weights were derived from the random-effects model with restricted maximum likelihood estimation, incorporating both within-study variance and between-study heterogeneity in the calculation.^32^ Heterogeneity of overall effect size was assessed using the *I*^2^ statistic; the proportion of variation across studies due to heterogeneity rather than chance, expressed as a percentage. Standard cutoffs for *I*^2^ were used, with 25%, 50%, and 75% indicating low, moderate, and high heterogeneity, respectively.^33^ To examine whether any study contributed disproportionately to the pooled effect size, leave-one-out sensitivity analyses were performed.^32^ Funnel plot visualization and Egger test were used to examine publication bias where possible.^34^

## Results

**Figure 1.**
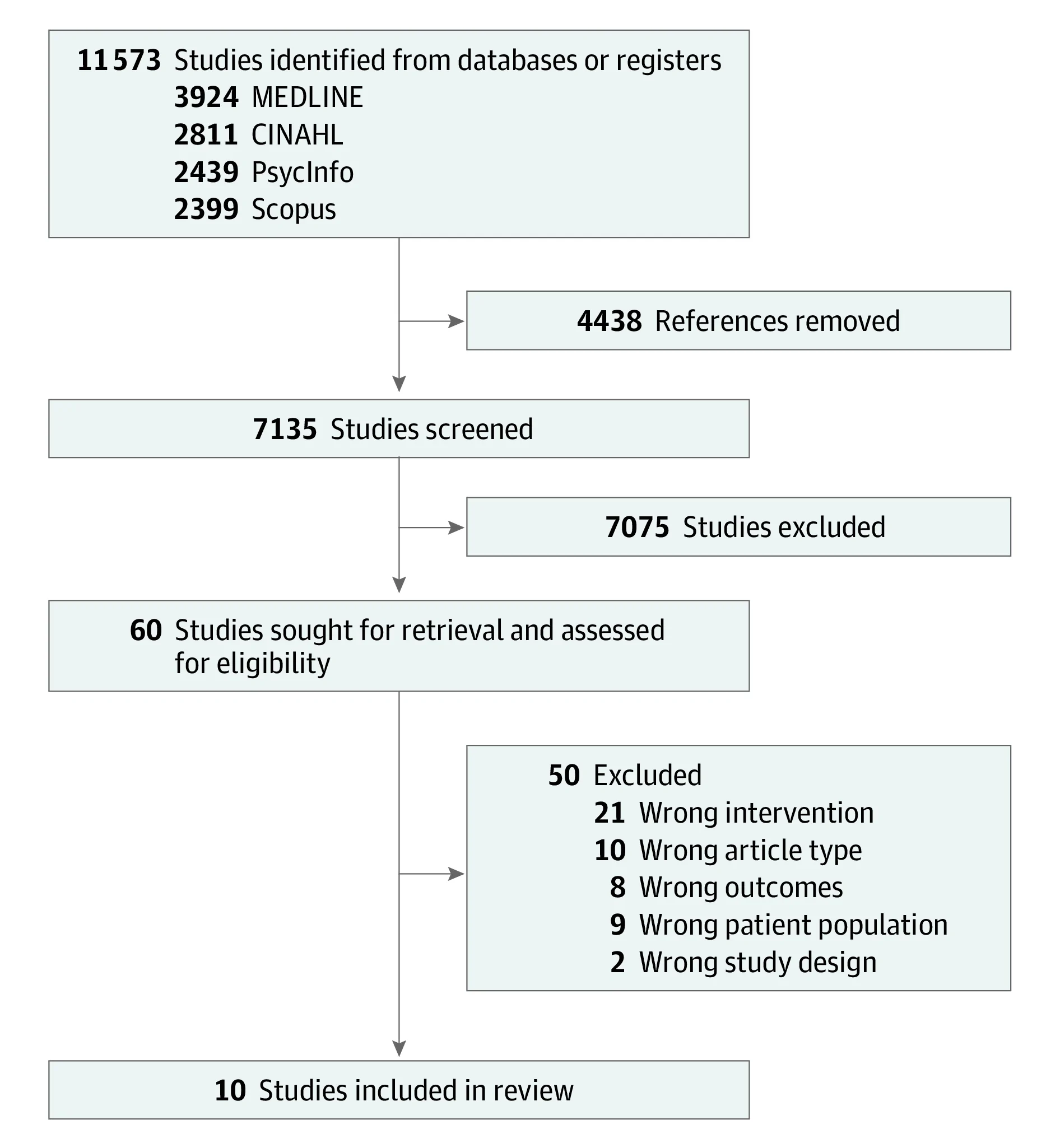
PRISMA Flow Diagram

Search results are summarized in Figure 1. The initial search retrieved unique 7136 studies for title and abstract screening after deduplication. Following title and abstract screening, 60 articles underwent full-text review, and of these, 50 studies were excluded, yielding 10 studies^35,36,37,38,39,40,41,42,43,44^ for data extraction and synthesis. No additional studies were identified by review of reference lists of included studies. Five studies^35,38,40,43,44^ were included in the meta-analysis.

### Study Characteristics

Study characteristics are detailed in Table 1. Ten studies^35,36,37,38,39,40,41,42,43,44^ based on 9 unique samples were included; 2 studies^39,41^ were derived from the same sample of adolescents. Five studies^35,38,39,40,44^ were randomized clinical trials (RCTs), and 5 studies^36,37,41,42,43^ used a nonrandomized experimental design. All studies were published from 2011 onward, and all were conducted in the US.

**Table 1.** Study and Intervention Characteristics

Source	Study design	Setting	Overall sample^a^	Treatment sample	Control sample	Treatment condition	Control condition	Treatment outcome (measure)	Risk of bias
Asarnow et al,^35^ 2011	RCT	ED	N = 181 Mean (SD) age, 14.7 (2.0) y; 69% female; 13% African American, 45% Hispanic, 33% White, 9% other	n = 89 Mean (SD) age, 14.8 (2.1) y; 66% female; 14% African American, 42% Hispanic, 35% White, 10% other	n = 92 Mean (SD) age, 14.6 (1.9 y); 72% female; 12% African American, 49% Hispanic, 32% White, 8% other	Intervention: family intervention for suicide prevention (youth and family crisis therapy session, included SPI); dosage: 1 session + phone calls	Intervention: enhanced TAU (TAU + linking to outpatient mental health treatment); dosage: NR	SI (DISC-IV; HASS^b^)^c^ SRB (DISC-IV; HASS^b^)^c^ SA (DISC-IV)^c^	Moderate
Bagatelas et al,^36^ 2022	Non-RCT	IU	N = 95 Mean (SD) age, 14.67 (1.76) y; 72.2% female; 1.39% Asian, 6.94% Black, 19.44% Hispanic/Latin, 70.14% White	NA	NA	Intervention: SPI; dosage: 1 session	NA	Re-presentation (percent re-hospitalized for SI/SRB within 6 mo)	Moderate
Cwik et al,^37^ 2016	Non-RCT (Pilot)	CMHC	N = 13 Mean (SD) age, 14.3 (2.2) y; 92% female; 100% Indigenous (Apache)	NA	NA	Intervention: new hope (novel intervention for Indigenous youth with recent suicide attempt, included SPI); dosage: 1-2 sessions	NA	SI (SIQ-Jr)	Moderate
Czyz et al,^38^ 2019	RCT (Pilot)	IU + OPC	N = 36 Mean (SD) age, 15.42 (1.36) y; 78.8% female; 8.3% African American or Black, 2.8% American Indian or Alaskan Native, 8.3% Asian, 5.6% Hispanic, 2.8% Native Hawaiian or Pacific Islander, 86.1% White	n = 18 Mean age, sex/gender, and ethnicity NR	n = 18 Mean age, sex/gender, and ethnicity NR	Intervention: MI-SafeCope (motivational interviewing + SPI) + TAU; dosage: 1 session +1 booster phone call	Intervention: TAU; dosage: 1 session	SI (C-SSRS)^c^ SRB (C-SSRS)^c^ SA (C-SSRS) Re-presentation (percent re-hospitalized for SI/SRB within 3 mo)^c^	Moderate
Czyz et al,^39^ 2021	RCT (Pilot SMART)	IU	N = 80 Mean (SD) age, 15.16 (1.35) y; 67.5% female; 6.3% African American or Black, 5.0% American Indian or Alaska Native, 5.0% Asian, 11.3% Hispanic, 1.4% Native Hawaiian or Other Pacific Islander, 83.8% White, 2.5% other	NR	NR	Intervention: MI-enhanced safety plan (motivational interview + SPI) + TAU + text support; dosage: 1 session; note: phase 2 of SMART ± booster phone call	Intervention: MI-enhanced safety plan (motivational interview + SPI) + TAU, no text-support; dosage: 1 session; note: phase 2 of SMART ± booster phone call	SI (C-SSRS) SRB (C-SSRS) SA (C-SSRS) Re-presentation (percent re-hospitalized for SI/SRB within 3 mo)	Moderate
Kennard et al,^40^ 2018	RCT (Pilot)	IU	N = 66 Mean (SD) age, 15.1 (1.5) y; 89.4% female; 77.3% White (other ethnicities NR)	n = 34 Mean (SD) age, 14.9 (1.9) y; 88.2% female; 2.9% Hispanic 79.4% White	n = 32 Mean (SD) age, 15.3 (1.4) y; 90.6% female; 0% Hispanic, 75.0% White	Intervention: as safe as possible (intervention informed by DBT and MI, included SPI); supplemented with phone app-based support (BRITE [Brite Technologies]), family sessions and bridging phone call; dosage: 2-5 sessions (median = 3)	Intervention: TAU; dosage: NR	SI (SIQ-Jr)^c^ SRB (C-SSRS) SA (C-SSRS)^d^	Moderate
May et al,^41^ 2023^e^	Non-RCT	IU + OPC	N = 80 Mean (SD) age, 15.2 (1.4) y; 69% female; 6.4% African American or Black, 5.1% American Indian or Alaska Native, 5.1% Asian, 1.3% Native Hawaiian or Other Pacific Islander, 83.3% White, 2.6% other	NA	NA	Intervention: see Czyz et al,^39^ 2021; dosage: NR	Intervention: see Czyz et al,^39^ 2021; dosage: NR	SI (single item assessing daily SI)	Low
Rengasamy and Sparks,^42^ 2019	Non-RCT (quasi- RCT)	IU	N = 142 Mean (SD) age, 15 (1.6) y; 70% female; 22% African American, 13% Asian, 1% Native American, 74% White	n = 72 Mean (SD) age, 15 (1.6) y; 67% female; 25% non-White	n = 70 Mean age NR; 73% female; 27% non-White	Intervention: SPI + multiple follow-up phone calls; dosage: single or multiple brief sessions within 7-d hospitalization +6 phone calls	Intervention: SPI + single follow-up phone call; dosage: single or multiple brief sessions within 7-d hospitalization +1 phone call	SRB (Columbia Classification Algorithm of Suicide Assessment)^d^ Re-presentation (Percent re-hospitalized for SI/SRB within 3 mo)^d^	Moderate
Wharff et al,^43^ 2012	Non-RCT (pilot)	ED	N = 250 Mean age, sex/gender, and ethnicity NR	n = 100 Mean (SD) age, 15.6 (1.45) y; 76% female; 2% Asian, 3% biracial, 16% Black, 11% Hispanic or Latino, 65% White, 3% other	n = 150 Mean (SD) age, 15.5 (1.47) y; 74% female; 2.7% Asian, 1.3% biracial, 17.3% Black, 11.0% Hispanic or Latino, 64.7% White, 4.0% other	Intervention: family-based crisis intervention (intervention informed by CBT, narrative therapy, and family systems therapy, included SPI); dosage: 1 session	Intervention: retrospective comparison group, no intervention received; dosage: None	Re-presentation (Percent re-hospitalized for SI/SRB within 6 mo)	High
Wharff et al,^44^ 2019	RCT	ED	N = 139 Mean (SD) age, 15.5 (1.4) y; 72% female; 3% Asian, 6% Black, 9% Latino, 18% multiracial, 66% White	n = 68 Mean (SD) age, 15.4 (1.3) y; 74% female; 4% Asian, 4% Black, 9% Latino, 21% multiracial, 62% White	n = 71 Mean (SD) age, 15.6 (1.5) y; 70% female; 1% Asian, 8% Black, 10% Latino, 15% multiracial, 70% White	Intervention: family-based crisis intervention (included SPI); dosage: 1 session	Intervention: TAU; dosage: 1 session	SRB (RLF-A)^d^ Re-presentation (percent re-hospitalized for SI/SRB within 6 mo)^c^	Moderate

Abbreviations: C-SSRS, Columbia Suicide Severity Rating Scale; CBT, cognitive behavioral therapy; CMHC, community mental health clinic; DBT, dialectical behavioral therapy; DISC-IV, National Institute for Mental Health Diagnostic Interview Schedule for Children Version IV; ED, emergency department; HASS, Harkavy Asnis Suicide Scale; IU, inpatient unit; MI, motivational interviewing; NA, not applicable; NR, not reported; OPC, outpatient psychiatric clinic; RCT, randomized controlled trial; RFL-A, Reasons for Living Inventory for Adolescents; SA, suicide attempt; SI, suicide ideation; SIQ-Jr, Suicide Ideation Questionnaire-Junior High School Version; SMART, sequential multiple assignment randomized trial; SPI, safety planning intervention; SRB, suicide-related behavior; TAU, treatment as usual.

^a^ Age in years; sex based on female/male binary; ethnicity categories not always mutually exclusive.

^b^ Outcome measure included in meta-analysis.

^c^ Outcome designated as secondary.

^d^ Outcome designated as primary.

^e^ Data derived from Czyz et al,^39^ 2021.

Overall, studies comprised 1002 adolescents between 10 and 19 years old (mean [SD] age, 15.0 [0.4] years; 76.0% female; 24.0% male). Only 2 studies^35,37^ included children under 12 years of age. The sex distribution of samples ranged from 66% to 92% female (mean [SD], 76.0% [9.9%]). In terms of racial distribution, 8 studies^36,38,39,40,41,42,43,44^ comprised samples that were predominantly White, 6 studies^36,38,39,40,41,42^ of which included more than 70% White participants. One sample^35^ was predominantly Hispanic, and 1 study^37^ included 100% Indigenous participants. Reporting of other demographic (eg, socioeconomic status) and clinical characteristics (eg, psychiatric illness) was inconsistent across studies.

### SPI Components

There was considerable variability across studies regarding SPI components and comprehensiveness (Table 2).^35,36,37,38,39,40,41,42,43,44^ Four studies^41,42,43,44^ did not provide information about the components that were implemented as part of the SPI. All 6 studies^35,36,37,38,39,40^ that described SPI components incorporated internal coping strategies (ie, those that do not require assistance from another person) and external coping strategies (eg, socialization or distraction). SPIs in 5 studies^35,36,38,39,40^ involved recognizing triggers or warning signs of crisis and identifying social contacts^36,37,38,39,40^ (ie, recording contact information for adults or friends the adolescents can reach out to when in crisis). Four studies^35,36,38,39^ specified that restricting access to lethal means was discussed and provided adolescents with contact information for professional support^36,38,39,40^ (eg, therapist; crisis call centers) as part of the SPI. Finally, 3 studies^35,36,40^ incorporated adolescent’s reason for living as part of the SPI. Across the 10 studies^35,36,37,38,39,40,41,42,43,44^ included, 8 studies^35,36,37,38,39,40,42,43,44^ described parent/caregiver involvement in developing the adolescent’s safety plan; the remaining 2 studies^36,41^ did not report whether parents were involved.

**Table 2.** Components of Safety Plan Interventions

Source	SPI components described?	Recognizing triggers of crisis	Internal coping strategies	External coping strategies	Identifying social contacts	Contact information for professional support	Restricting access to lethal means	Reasons for living	Parent involvement
Asarnow et al,^35^ 2011	Yes	E/R	E/R	E/R	NR	NR	E/R	E/R	E/R
Bagatelas et al,^36^ 2022	Yes	E/R	E/R	E/R	E/R	E/R	E/R	E/R	NR
Cwik et al,^37^ 2016	Yes	NR	E/R	E/R	E/R	NR	NR	NR	E/R
Czyz et al,^38^ 2019	Yes	E/R	E/R	E/R	E/R	E/R	E/R	NR	E/R
Czyz et al,^39^ 2021	Yes	E/R	E/R	E/R	E/R	E/R	E/R	NR	E/R
Kennard et al,^40^ 2018	Yes	E/R	E/R	E/R	E/R	E/R	NR	E/R	E/R
May et al,^41^ 2023	No	NR	NR	NR	NR	NR	NR	NR	
Rengasamy and Sparks,^42^ 2019	No	NR	NR	NR	NR	NR	NR	NR	E/R
Wharff et al,^43^ 2012	No	NR	NR	NR	NR	NR	NR	NR	E/R
Wharff et al,^44^ 2019	No	NR	NR	NR	NR	NR	NR	NR	E/R

Abbreviation: E/R, endorsed/reported; NR, not reported; SPI, safety plan intervention.

### Quality Appraisal

Item-level responses to the JBI Critical Appraisal Tools are provided in eTables 2 and 3 in Supplement 1. All RCTs (n = 5) were rated as having a moderate risk of bias. For studies using nonrandomized experimental designs (n = 5), 1 study^41^ was rated as having low risk of bias, 1 study^43^ was rated as having high risk of bias, and the remaining 3 studies^36,37,42^ were rated as having moderate risk of bias.

### Meta-Analysis

**Figure 2.**
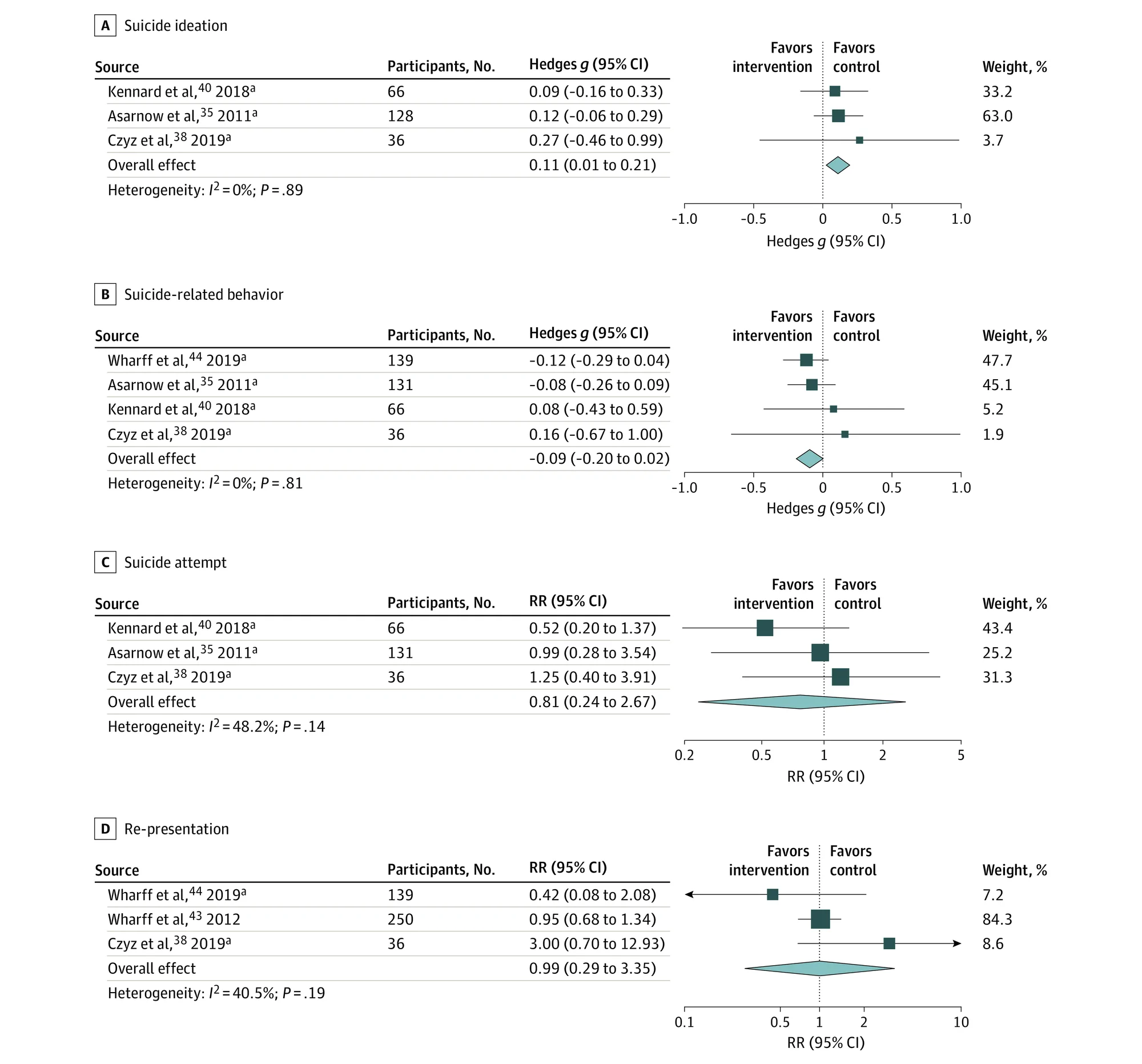
Forest Plots of the Association Between Safety Planning Interventions (SPIs) and Suicide-Related Outcomes Forest plots of the association between SPIs and suicide ideation (A), suicide-related behavior (SRB) (B), suicide attempts (C), and re-presentation (D). Suicide ideation and SRB estimates were calculated using Hedges *g* and suicide attempt and re-presentation were calculated using risk ratio (RR). ^a^Randomized clinical trial.

Of the 10 studies^35,36,37,38,39,40,41,42,43,44^ meeting inclusion criteria, 5 studies^35,38,40,43,44^ reported data available for meta-analyses. Studies assessing SI (n = 3), SRB (n = 4), suicide attempts (n = 3), and re-presentation (n = 3) had sample sizes ranging from 36 to 250 participants. More specifically, SI was designated as a primary outcome in zero studies and designated as a secondary outcome in 3 studies.^35,38,40^ SRB was designated as a primary outcome in 1 study^44^ and a secondary outcome in 2 studies.^35,38^ SA was designated as a primary outcome in 1 study^40^ and a secondary outcome in 1 study.^35^ Re-presentation to acute care settings was designated as the primary outcome in zero studies and a secondary outcome in 2 studies.^38,44^ One study^43^ did not report which outcomes were designated as primary or secondary. The effect of SPIs on these outcomes was estimated using random-effects models and displayed in Figure 2. No significant associations were observed between SPIs and SI (Hedges *g* = 0.11; 95% CI, 0.01-0.21) (Figure 2A), SRB (Hedges *g* = −0.09; 95% CI, −0.20 to 0.02) (Figure 2B), SA (RR, 0.81; 95% CI, 0.24-2.67) (Figure 2C), or re-presentation (RR, 0.99; 95% CI, 0.29-3.35) (Figure 2D). Heterogeneity was low and nonsignificant in all meta-analyses. The number of studies (n < 10) did not allow for assessment of publication bias. Sensitivity analyses were conducted to assess the robustness of the association between safety planning and outcomes of interest based on study design and control condition. Results for all outcomes were consistent with those of the full sample after removal of non–RCT-designed studies and on inclusion of studies that involved any SPI component in the control condition^39,42^ (eFigure in Supplement 1). Leave-one-out sensitivity analyses were also conducted, with results again consistent with those of the full sample. As the number of studies was small and heterogeneity was low, meta-regression was not conducted.^32^

## Discussion

To our knowledge, this was the first meta-analysis of the effectiveness of safety planning as a standalone treatment for children and adolescents at risk for suicide. The focus of this review was to estimate the effects of safety planning on suicide-related outcomes (ie, SI, SRB, and SA) and re-presentation to acute care settings, which are known predictors of recurrent suicide attempts and death from suicide.^45,46^ We found no association between SPI and reduction in SI, SRB, SA, or re-presentation to acute care settings at follow-up 1 to 6 months after SPI. Variability across studies, in terms of the specific SPI components, was also noted.

Research demonstrating the effectiveness of SPIs for adults with SITBs has resulted in their widespread implementation in adolescent settings.^47^ Results of this meta-analysis, however, suggest that in contrast to findings in adults, SPIs do not decrease SITBs for adolescents in crisis. A differential effectiveness of treatment by patient age has similarly been noted for other interventions, including pharmacological treatments, with potentially harmful effects for pediatric populations^48,49^ Differences in SITB presentations and precipitants may underlie, in part, the differential effects of SPIs observed for adolescents and adults. For example, in comparison with adults, adolescents who present to emergency departments for SAs have a higher number of previous attempts, are more likely to use nonlethal means and more frequently experience interpersonal conflict as the precipitant to a suicide attempt vs financial or illness-related stressors in adult samples.^50^ SPIs, as currently designed, may not necessarily address these adolescent-specific drivers, impacting intervention effectiveness for this age group. Key developmental adaptions may enhance the effectiveness of SPIs for adolescents.^51^ For example, parent or family involvement may be an important modification^44^ because of the prominent role families have during this developmental stage and the apparent link between family functioning and adolescent SITB.^52,53^ Although most included studies did involve parents in the SPI, the nature of involvement varied, and no formal evaluation of parent involvement was conducted. Based on study-level results, adolescents who received interventions that were family-based had lower rates of re-presentation^44^ and were more likely to be linked to outpatient mental health services after discharge^35^ compared with adolescents who received treatment as usual. In addition, increased follow-up phone contact with patients in the week after discharge^42^ and higher quality safety plans (ie, incorporating all key SPI components)^36^ may also be linked to improved outcomes.

In addition to developmental adaptations and considerations, differences in effectiveness of SPIs among adolescent vs adult samples may also partly stem from the relatively smaller and more nascent evidence base for adolescents. Of the included studies, 5 were pilot trials,^37,38,39,40,43^ with insufficient power to detect small treatment effect sizes. Only 3 studies^35,42,44^ had adequate-sized samples to compare treatment effects across groups; 2 of these studies^35,44^ used true randomization, and neither found significant between-group differences in suicide-related outcomes. Comparatively, meta-analytic findings^16^ of studies involving adults were derived in part from 2 well-powered RCTs,^54,55^ and 2 nonrandomized trials with large samples (ie, n* *>1000 each).^56,57^ In controlled trials with children and adolescents,^35,38,39,40,42,43,44^ control conditions were often vaguely defined and sometimes involved elements of safety planning to varying degrees, although results were consistent whether these studies were removed (Figure 2) or included (eFigure in Supplement 1). In contrast, SPIs with adults were commonly compared with control conditions that involved risk screening, assessment, or case management, and infrequently included safety planning components (eg, coping strategies).^16^ The presumptive and widespread adoption of SPIs as standard care for adolescents with SITBs is also an important consideration for future research, resulting in increased likelihood that treatment-as-usual control conditions will involve at least some SPI components.

### Strengths and Limitations

There are several strengths of this review. First, we focused on studies that examined SPIs as a standalone treatment, excluding studies where safety planning was an element of a multicomponent intervention (eg, safety planning session within cognitive behavior therapy). This provides an ecologically valid indication of the effectiveness of SPIs in real-world settings, where safety planning is typically delivered in a single session with minimal or no follow-up (eg, during emergency department visits). This review also documented the key SPI components initially described by Stanley and Brown,^14^ which captured the nuances and variability of safety plans that are often overlooked.

Several limitations merit mention. First, the search was restricted to English language, and further evidence may be available in non-English publications. Although heterogeneity of effects was noted, potential moderators of treatment effect, including methodological factors (eg, study design),^16^ treatment-related factors (eg, clinician training),^15,36^ and patient-related factors (eg, age, gender) could not be examined. Absence of detailed demographic data precluded examination of effectiveness of SPIs by socioeconomic risk and race and ethnic group. Lastly, all studies were conducted in the US. Thus, the potential effectiveness of SPIs in non-US health care settings, including those that administer a publicly funded health care system, remains undetermined.

## Conclusions

In summary, findings of this systematic review and meta-analysis indicate that the evidence base for SPIs in adolescents was small, and SPIs were not associated with reductions in SITB, including SAs, or re-presentation to acute care settings. Given escalating rates of SITB in adolescents and increased need for treatment,^1,3,6,7^ SPIs have been widely adopted as feasible and implementable suicide prevention interventions in acute care settings. However, there is a need for better understanding of which SPI components are effective, which adolescents are most likely to respond to single-session treatments,^58^ and the specific training considerations needed to ensure clinicians are providing adolescents with high-quality SPIs.^59^ Thus, the opportunity to advance the evidence-base for single-session suicide prevention interventions for adolescents is vast. Well-designed, adequately powered RCTs that specify SPI elements, have clearly defined control conditions, and are conducted in representative study samples are needed to determine how to most effectively conduct safety planning with children and adolescents at increased risk for suicide and whether safety planning can effectively serve as a standalone intervention in this population or requires integration with other suicide prevention strategies.

## Notes

Supplement 1. **eTable 1.** Search Strategy **eTable 2.** JBI Quality Appraisal Ratings for RCTs **eTable 3.** JBI Quality Appraisal Ratings for Non-RCTs **eFigure.** Association Between SPIs and Suicide-Related Outcomes, Inclusive of Studies Containing SPIs in the Control Condition

Supplement 2.
Data Sharing Statement.
