# Supplementary material for: Safety Planning Interventions for Suicide Prevention in Children and Adolescents: A Systematic Review and Meta-Analysis
Source: JAMA Pediatr. 2025 May 19;179(8):886–95. doi: 10.1001/jamapediatrics.2025.1012 (PMC12090068; doi:10.1001/jamapediatrics.2025.1012)
Supplement: Supplement 1. — eTable 1. PRISMA Checklist and Abstracts Checklist eTable 2. Search Strategy eTable 3. JBI Quality Appraisal Ratings for RCTs eTable 4. JBI Quality Appraisal Ratings for Non-RCTs eFigure. Association Between SPIs and Suicide-Related Outcomes, Inclusive of Studies Containing SPIs in the Control Condition [file jamapediatr-e251012-s001.pdf]

Supplementary Online Content

Albaum C, Irwin SH, Muha J, et al. Safety planning interventions for suicide prevention in children and adolescents: a systematic review and meta-analysis. *JAMA Pediatr*. Published online May 19, 2025. doi:10.1001/jamapediatrics.2025.1012

eTable 1. PRISMA Checklist and Abstracts Checklist

eTable 2. Search Strategy

eTable 3. JBI Quality Appraisal Ratings for RCTs

eTable 4. JBI Quality Appraisal Ratings for Non-RCTs

eFigure. Association Between SPIs and Suicide-Related Outcomes, Inclusive of Studies Containing SPIs in the Control Condition

This supplementary material has been provided by the authors to give readers additional information about their work.

© 2025 Albaum C et al. *JAMA Pediatrics.*

eTable 1. **PRISMA Checklist and Abstracts Checklist**

Section and Topic	Item #	Checklist item	Location where item is reported
**TITLE**			
Title	1	Identify the report as a systematic review.	Page 1
**ABSTRACT**			
Abstract	2	See the PRISMA 2020 for Abstracts checklist.	See below
**INTRODUCTION**			
Rationale	3	Describe the rationale for the review in the context of existing knowledge.	Page 4
Objectives	4	Provide an explicit statement of the objective(s) or question(s) the review addresses.	Page 5-6
**METHODS**			
Eligibility criteria	5	Specify the inclusion and exclusion criteria for the review and how studies were grouped for the syntheses.	Page 7
Information sources	6	Specify all databases, registers, websites, organisations, reference lists and other sources searched or consulted to identify studies. Specify the date when each source was last searched or consulted.	Page 6
Search strategy	7	Present the full search strategies for all databases, registers and websites, including any filters and limits used.	Supplemental Table 2
Selection process	8	Specify the methods used to decide whether a study met the inclusion criteria of the review, including how many reviewers screened each record and each report retrieved, whether they worked independently, and if applicable, details of automation tools used in the process.	Page 7
Data collection process	9	Specify the methods used to collect data from reports, including how many reviewers collected data from each report, whether they worked independently, any processes for obtaining or confirming data from study investigators, and if applicable, details of automation tools used in the process.	Page 7
Data items	10a	List and define all outcomes for which data were sought. Specify whether all results that were compatible with each outcome domain in each study were sought (e.g. for all measures, time points, analyses), and if not, the methods used to decide which results to collect.	Table 1
	10b	List and define all other variables for which data were sought (e.g. participant and intervention characteristics, funding sources). Describe any assumptions made about any missing or unclear information.	Table 1
Study risk of bias assessment	11	Specify the methods used to assess risk of bias in the included studies, including details of the tool(s) used, how many reviewers assessed each study and whether they worked independently, and if applicable, details of automation tools used in the process.	Page 8
Effect measures	12	Specify for each outcome the effect measure(s) (e.g. risk ratio, mean difference) used in the synthesis or presentation of results.	Page 8
Synthesis	13a	Describe the processes used to decide which studies were eligible for each synthesis (e.g. tabulating the study intervention	Page 8, 11

© 2025 Albaum C et al. *JAMA Pediatrics.*

Section and Topic	Item #	Checklist item	Location where item is reported
methods		characteristics and comparing against the planned groups for each synthesis (item #5)).	
	13b	Describe any methods required to prepare the data for presentation or synthesis, such as handling of missing summary statistics, or data conversions.	Page 8
	13c	Describe any methods used to tabulate or visually display results of individual studies and syntheses.	Page 8, Figure 2
	13d	Describe any methods used to synthesize results and provide a rationale for the choice(s). If meta-analysis was performed, describe the model(s), method(s) to identify the presence and extent of statistical heterogeneity, and software package(s) used.	Page 8
	13e	Describe any methods used to explore possible causes of heterogeneity among study results (e.g. subgroup analysis, meta-regression).	Page 8-9
	13f	Describe any sensitivity analyses conducted to assess robustness of the synthesized results.	Page 9
Reporting bias assessment	14	Describe any methods used to assess risk of bias due to missing results in a synthesis (arising from reporting biases).	N/A
Certainty assessment	15	Describe any methods used to assess certainty (or confidence) in the body of evidence for an outcome.	N/A
**RESULTS**			
Study selection	16a	Describe the results of the search and selection process, from the number of records identified in the search to the number of studies included in the review, ideally using a flow diagram.	Page 9, Figure 1
	16b	Cite studies that might appear to meet the inclusion criteria, but which were excluded, and explain why they were excluded.	N/A
Study characteristics	17	Cite each included study and present its characteristics.	Page 9-10, Table 1
Risk of bias in studies	18	Present assessments of risk of bias for each included study.	Page 10-11, Supplemental Tables 3 and 4
Results of individual studies	19	For all outcomes, present, for each study: (a) summary statistics for each group (where appropriate) and (b) an effect estimate and its precision (e.g. confidence/credible interval), ideally using structured tables or plots.	Figure 2
Results of syntheses	20a	For each synthesis, briefly summarise the characteristics and risk of bias among contributing studies.	Page 9-11
	20b	Present results of all statistical syntheses conducted. If meta-analysis was done, present for each the summary estimate and its precision (e.g. confidence/credible interval) and measures of statistical heterogeneity. If comparing groups, describe the direction of the effect.	Page 11-12, Figure 2

© 2025 Albaum C et al. *JAMA Pediatrics.*

Section and Topic	Item #	Checklist item	Location where item is reported
	20c	Present results of all investigations of possible causes of heterogeneity among study results.	Page 12
	20d	Present results of all sensitivity analyses conducted to assess the robustness of the synthesized results.	Page 11-12
Reporting biases	21	Present assessments of risk of bias due to missing results (arising from reporting biases) for each synthesis assessed.	N/A
Certainty of evidence	22	Present assessments of certainty (or confidence) in the body of evidence for each outcome assessed.	N/A
**DISCUSSION**			
Discussion	23a	Provide a general interpretation of the results in the context of other evidence.	Page 12-14
	23b	Discuss any limitations of the evidence included in the review.	Page 12-14
	23c	Discuss any limitations of the review processes used.	Page 14
	23d	Discuss implications of the results for practice, policy, and future research.	Page 15
**OTHER INFORMATION**			
Registration and protocol	24a	Provide registration information for the review, including register name and registration number, or state that the review was not registered.	Page 6
	24b	Indicate where the review protocol can be accessed, or state that a protocol was not prepared.	Page 6
	24c	Describe and explain any amendments to information provided at registration or in the protocol.	N/A
Support	25	Describe sources of financial or non-financial support for the review, and the role of the funders or sponsors in the review.	N/A
Competing interests	26	Declare any competing interests of review authors.	N/A
Availability of data, code and other materials	27	Report which of the following are publicly available and where they can be found: template data collection forms; data extracted from included studies; data used for all analyses; analytic code; any other materials used in the review.	N/A

From: Page MJ, McKenzie JE, Bossuyt PM, Boutron I, Hoffmann TC, Mulrow CD, et al. The PRISMA 2020 statement: an updated guideline for reporting systematic reviews. BMJ 2021;372:n71. doi: 10.1136/bmj.n71.
This work is licensed under CC BY 4.0. To view a copy of this license, visit https://creativecommons.org/licenses/by/4.0/

© 2025 Albaum C et al. *JAMA Pediatrics.*

PRISMA 2020 for Abstracts Checklist

Section and Topic	Item #	Checklist item	Reported (Yes/No)
**TITLE**			
Title	1	Identify the report as a systematic review.	Yes
**BACKGROUND**			
Objectives	2	Provide an explicit statement of the main objective(s) or question(s) the review addresses.	Yes
**METHODS**			
Eligibility criteria	3	Specify the inclusion and exclusion criteria for the review.	Yes
Information sources	4	Specify the information sources (e.g. databases, registers) used to identify studies and the date when each was last searched.	Yes
Risk of bias	5	Specify the methods used to assess risk of bias in the included studies.	Yes
Synthesis of results	6	Specify the methods used to present and synthesise results.	Yes
**RESULTS**			
Included studies	7	Give the total number of included studies and participants and summarise relevant characteristics of studies.	Yes
Synthesis of results	8	Present results for main outcomes, preferably indicating the number of included studies and participants for each. If meta-analysis was done, report the summary estimate and confidence/credible interval. If comparing groups, indicate the direction of the effect (i.e. which group is favoured).	Yes
**DISCUSSION**			
Limitations of evidence	9	Provide a brief summary of the limitations of the evidence included in the review (e.g. study risk of bias, inconsistency and imprecision).	Yes
Interpretation	10	Provide a general interpretation of the results and important implications.	Yes
**OTHER**			
Funding	11	Specify the primary source of funding for the review.	N/A
Registration	12	Provide the register name and registration number.	Yes

From: Page MJ, McKenzie JE, Bossuyt PM, Boutron I, Hoffmann TC, Mulrow CD, et al. The PRISMA 2020 statement: an updated guideline for reporting systematic reviews. BMJ 2021;372:n71. doi: 10.1136/bmj.n71. This work is licensed under CC BY 4.0. To view a copy of this license, visit https://creativecommons.org/licenses/by/4.0/

© 2025 Albaum C et al. *JAMA Pediatrics.*

eTable 2. Search strategy

#	Searches
**Ovid MEDLINE(R) Epub Ahead of Print and In-Process, In-Data-Review & Other Non-Indexed Citations**	
1	Child/ or Adolescent/ or (child* or teen* or "teen-age*" or Youth? or "Young people" or "Young person?" or juvenile* or highschool* or "high-school*" or "school age*" or p?ediatric*).mp.
2	Suicide Prevention/ or ((suicid* or self-harm* or self-injur*) adj2 (intervention* or reduc* or prevent* or awareness or SPI)).mp.
3	Crisis Intervention/ or ((cris?s or coping or cope*) adj2 (plan* or strateg* or manage* or intervention* or program* or respon* or treatment? or mechanism?)).mp.
4	*Harm Reduction/ or (Harm adj2 reduc*).mp.
5	*Safety Management/ or ((safety or risk*) adj (plan* or strateg* or manage* or respon*)).mp.
6	or/2-5
7	*Suicide/ or Suicide, Attempted/ or Suicidal Ideation/ or ((suicid* adj (attempt* or ideat* or plan* or behavio?r*)) or parasuicid* or "para-suicid*").mp.
8	*Self-Injurious Behavior/ or ((Self-Injur* or self-harm* or self-destructive) adj2 (attempt* or plan* or behavio?r* or intentional* or deliberate* or hospitaliz* or hospitalis* or institutionali* or "hospital admission" or "patient? Admission")).mp.
9	7 or 8
10	1 and 6 and 9
11	Limt 10 to yr= “2008-Current”
12	Remove duplicates from 11
**APA PsycInfo 1806 to March Week 2 2024**	
1	Child psychology/ or Adolescent psychology/ or (child* or teen* or "teen-age*" or Youth? or "Young people" or "Young person?" or juvenile* or highschool* or "high-school*" or "school age*" or p?ediatric*).mp.
2	Suicide Prevention/ or ((suicid* or self-harm* or self-injur*) adj2 (intervention* or reduc* or prevent* or awareness or SPI)).mp.
3	Crisis Intervention/ or ((cris?s or coping or cope*) adj2 (plan* or strateg* or manage* or intervention* or program* or respon* or treatment? or mechanism?)).mp.
4	Harm Reduction/ or (Harm adj2 reduc*).mp.
5	Risk Management/ or ((safety or risk*) adj (plan* or strateg* or manage* or respon*)).mp.
6	or/2-5
7	*Suicide/ or Attempted suicide/ or Suicidal Ideation/ or ((suicid* adj (attempt* or ideat* or plan* or behavio?r*)) or parasuicid* or "para-suicid*").mp.
8	Nonsuicidal self-injury/ or ((Self-Injur* or self-harm* or self-destructive) adj2 (attempt* or plan* or behavio?r* or intentional* or deliberate* or hospitaliz* or hospitalis* or institutionali* or "hospital admission" or "patient? Admission")).mp.
9	7 or 8
10	1 and 6 and 9
11	Limit 10 to yr=“2008-Current”
12	Remove duplicates from 11
**CINAHL Plus with Full Text**	
S1	MH Child or MH Adolescent or TX (child* or teen* or "teen-age*" or Youth? or "Young people" or "Young person?" or juvenile* or highschool* or "high-school*" or "school age*" or p?ediatric*)
S2	MM Suicide Prevention or ((suicid* or self-harm* or self-injur*) W2 (intervention* or reduc* or prevent* or awareness or SPI))
S3	MM Crisis Intervention or TX ((cris?s or coping or cope*) W2 (plan* or strateg* or manage* or intervention* or program* or respon* or treatment? or mechanism?))
S4	MM Harm Reduction or TX (Harm W2 reduc*)
S5	MM Risk Management or TX ((safety or risk*) W1 (plan* or strateg* or manage* or respon*))
S6	S2 OR S3 OR S4 OR S5
S7	S1 AND S6

© 2025 Albaum C et al. *JAMA Pediatrics.*

#	Searches
S8	MM Suicide or MH Suicide, attempted or MH Suicidal Ideation or TX ((suicid* W1 (attempt* or ideat* or plan* or behavio?r*)) or parasuicid* or "para-suicid*")
S9	MM Self-injurious behavior or TX ((Self-Injur* or self-harm* or self-destructive) W2 (attempt* or plan* or behavio?r* or intentional* or deliberate* or hospitaliz* or hospitalis* or institutionali* or "hospital admission" or "patient? Admission"))
S10	S8 OR S9
S11	S7 AND S10
**Scopus**	
1	Title-Abs-Key (child* or teen* or "teen-age*" or Youth? or "Young people" or "Young person?" or juvenile* or highschool* or "high-school*" or "school age*" or p?ediatric*)
2	Title-Abs-Key ((suicid* or self-harm* or self-injur*) W/2 (intervention* or reduc* or prevent* or awareness or SPI))
3	Title-Abs-Key ((cris?s or coping or cope*) W/2 (plan* or strateg* or manage* or intervention* or program* or respon* or treatment? or mechanism?))
4	Title-Abs-Key (Harm W/2 reduc*)
5	Title-Abs-Key ((safety or risk*) W/1 (plan* or strateg* or manage* or respon*))
6	Line 2 OR Line 3 OR Line 4 OR Line 5
7	Title-Abs-Key ((suicid* W/1 (attempt* or ideat* or plan* or behavio?r*)) or parasuicid* or "para-suicid*")
8	Title-Abs-Key ((Self-Injur* or self-harm* or self-destructive) W/2 (attempt* or plan* or behavio?r* or intentional* or deliberate* or hospitaliz* or hospitalis* or institutionali* or "hospital admission" or "patient? Admission"))
9	Line 7 OR 8
10	Line 1 AND Line 6 AND Line 9

© 2025 Albaum C et al. *JAMA Pediatrics.*

eTable 3. **JBI Quality appraisal ratings for RCTs**

	SELECTION AND ALLOCATION			ADMINISTRATION OF INTERVENTION			ASSESSMENT, DETECTION, AND OUTCOME MEASUREMENT			PARTICIPANT RETENTION	STATISTICAL CONCLUSION VALIDITY		
**Authors**	**1**	**2**	**3**	**4**	**5**	**6**	**7**	**8**	**9**	**10**	**11**	**12**	**13**
Asarnow et al.	Yes	Yes	Yes	No	No	Yes	Outcome 1: SI (DISC-IV)						Yes
							No	Yes	Yes	No	No	Unclear	
							Outcome 2: SRB (DISC-IV)						
							No	Yes	Yes	No	No	Yes	
							Outcome 3: SI (HASS)						
							No	Yes	Yes	No	No	Yes	
							Outcome 4: SRB (HASS)						
							No	Yes	Yes	No	No	Yes	
Czyz et al.	Yes	Yes	Unclear	No	No	Yes	Outcome 1: SA (Frequency)						Yes
							Yes	Yes	Yes	No	Unclear	No	
							Outcome 2: SI (C-SSRS)						
							No	Yes	Unclear	No	Unclear	Unclear	
							Outcome 3: Re-presentation (Frequency)						
							Yes	Yes	Yes	No	Unclear	No	
Czyz et al.	Yes	Unclear	Unclear	No	No	Yes	Outcome 1: SI (C-SSRS)						No
							No	Yes	Unclear	No	Unclear	Yes	
							Outcome 2: Daily SI (C-SSRS)						
							No	Yes	Unclear	No	Unclear	Yes	
							Outcome 3: SRB (C-SSRS)						
							No	Yes	Unclear	No	No	Yes	
							Outcome 4: Suicide attempt (Frequency)						
							Unclear	Yes	Yes	No	No	Yes	

© 2025 Albaum C et al. *JAMA Pediatrics.*

	SELECTION AND ALLOCATION			ADMINISTRATION OF INTERVENTION			ASSESSMENT, DETECTION, AND OUTCOME MEASUREMENT			PARTICIPANT RETENTION	STATISTICAL CONCLUSION VALIDITY		
							Outcome 5: Re-presentation (Frequency)						
							Unclear	Yes	Unclear	No	No	Yes	
Kennard et al.	Yes	Yes	Yes	No	No	Yes	Outcome 1: SI (SIQ-Jr)						Yes
							No	Yes	Yes	Yes	Unclear	Yes	
							Outcome 2: SRB (C-SSRS)						
							Yes	Yes	Yes	No	Unclear	Yes	
							Outcome 3: Suicide attempt (Frequency)						
							Unclear	Yes	Yes	No	Unclear	Yes	
Wharff et al.	Unclear	Yes	Yes	No	No	Yes	Outcome 1: SRB (RFL-A)						Yes
							No	Yes	Yes	Yes	No	Yes	
							Outcome 2: Re-presentation (Inpatient)						
							No	Yes	Unclear	Yes	No	Yes	
							Outcome 3: Re-presentation (1 Month)						
							No	Yes	Unclear	Yes	No	Yes	

Legend:
1. Was true randomization used for assignment of participants to treatment group?
2. Was allocation of treatment groups concealed?
3. Were treatment groups similar at baseline?
4. Were participants blind to treatment assignment?
5. Were those delivering the treatment blind to assignment?
6. Were treatment groups treated identically other than the intervention of interest?
7. Were outcome assessors blind to treatment assignment? (Specify outcome)
8. Were outcomes measured in the same way for treatment groups?
9. Were outcomes measured in a reliable way?
10. Was follow up complete and if not, were differences between groups in terms of their follow up adequately described and analyzed?
11. Were participants analyzed in the groups to which they were randomized?
12. Was appropriate statistical analysis used?
13. Was the trial design appropriate and any deviations from the standard RCT design (individual randomization, parallel groups) accounted for in the conduct and analysis of the trial?

© 2025 Albaum C et al. *JAMA Pediatrics.*

***eTable 4.* JBI Quality appraisal ratings for non-RCT**

Authors	1	2	3	4	5	6	7	8	9
Bagatelas et al.	Yes	Unclear	Yes	No	No	No	Yes	Yes	Yes
Cwik et al.	Yes	Yes	Yes	No	Yes	No	Yes	Yes	No
May et al.	No	Yes	Yes	No	Yes	Yes	Yes	Yes	Yes
Rengasamy et al.	No	Yes	Yes	Yes	Unclear	Yes	Yes	Unclear	Yes
Wharff et al.	Yes	Unclear	No	Yes	No	No	No	Unclear	No

Legend:
1. Is it clear what is the 'cause' and what is the 'effect' (i.e., there is no confusion about which variable comes first)?
2. Were the participants included in any comparisons similar?
3. Were the participants included in any comparisons receiving similar treatment/care, other than the exposure or intervention of interest?
4. Was there a control group?
5. Were there multiple measurements of the outcome both pre and post the intervention/exposure?
6. Was follow up complete and if not, were differences between groups in terms of their follow up adequately described and analyzed?
7. Were the outcomes of participants included in any comparisons measured in the same way?
8. Were outcomes measured in a reliable way?
9. Was appropriate statistical analysis used?

© 2025 Albaum C et al. *JAMA Pediatrics.*

eFigure. **Association between SPIs and suicide-related outcomes, inclusive of studies containing SPIs in the control condition**

a.

Suicide Ideation

Source	Hedges' g (95% CI)	N	Weight
Czyz 2021[(39) a]	-0.25 (-0.99; 0.50)	80	3.4%
Kennard 2018[(40) a]	0.09 (-0.16; 0.33)	66	32.1%
Asarnow 2011[(35) a]	0.12 (-0.06; 0.29)	128	60.9%
Czyz 2019[(38) a]	0.27 (-0.46; 0.99)	36	3.6%
**Overall Effect**	**0.10 (-0.03; 0.23)**		

Heterogeneity: I^2 = 0.0%, p = 0.78

[a] Randomized controlled trial

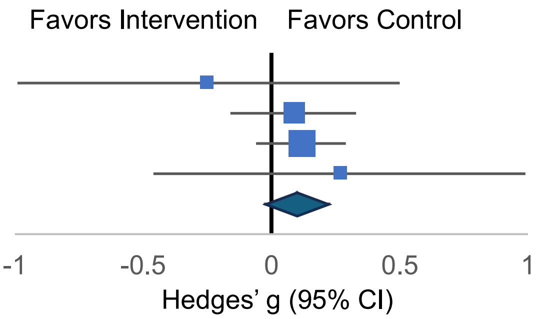

b.

Suicide-Related Behaviors

Source	Hedges' g (95% CI)	N	Weight
Rengasamy 2019[(42) a]	-0.70 (-1.35; -0.06)	142	3.1%
Czyz 2021[(39) a]	-0.62 (-1.40; 0.17)	80	2.1%
Wharff 2019[(44) a]	-0.12 (-0.29; 0.04)	139	45.3%
Asarnow 2011[(35) a]	-0.08 (-0.26; 0.09)	131	42.8%
Kennard 2018[(40) a]	0.08 (-0.43; 0.59)	66	4.9%
Czyz 2019[(38) a]	0.16 (-0.67; 1.00)	36	1.8%
**Overall Effect**	**-0.12 (-0.28; 0.04)**		

Heterogeneity: I^2 = 15.0%, p = 0.32

[a] Randomized controlled trial

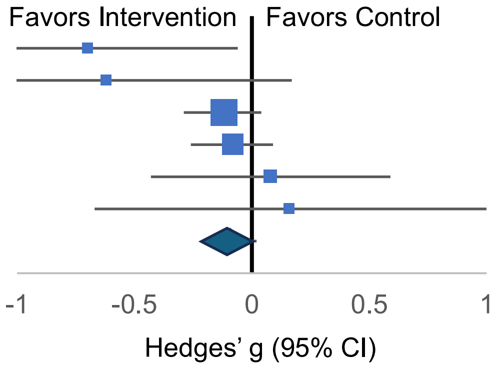

c.

Suicide Attempt

Source	RR (95% CI)	N	Weight
Czyz 2021[(39) a]	0.33 (0.07; 1.37)	66	20.0%
Kennard 2018[(40) a]	0.52 (0.20; 1.37)	66	33.5%
Asarnow 2011[(35) a]	0.99 (0.28; 3.54)	131	25.2%
Czyz 2019[(38) a]	3.00 (0.70; 12.93)	36	21.3%
**Overall Effect**	**0.81 (0.20; 3.37)**		

Heterogeneity: I^2 = 43.1%, p = 0.15

[a] Randomized controlled trial

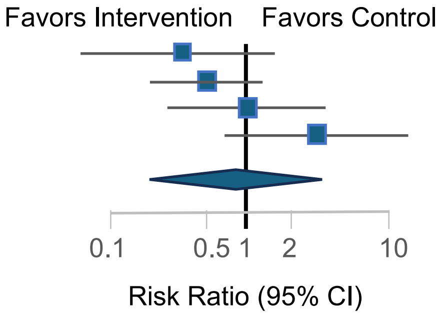

d.

Re-presentation

Source	RR (95% CI)	N	Weight
Wharff 2019[(44) a]	0.42 (0.08; 2.08)	139	3.2%
Rengasamy 2019[(42) a]	0.82 (0.40; 1.71)	142	15.3%
Czyz 2021[(39) a]	0.86 (0.29; 2.56)	80	6.8%
Wharff 2012[(43)]	0.95 (0.68; 1.34)	250	70.9%
Czyz 2019[(38) a]	3.00 (0.70; 12.93)	36	3.8%
**Overall Effect**	**0.94 (0.64; 1.38**		

Heterogeneity: I^2 = 0%, p = 0.47

[a] Randomized controlled trial

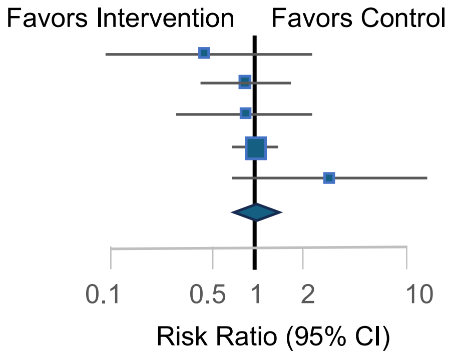

© 2025 Albaum C et al. *JAMA Pediatrics.*
