# Supplementary material for: Safety Planning Interventions for Suicide Prevention in Children and Adolescents: A Systematic Review and Meta-Analysis
Source: JAMA Pediatr. 2025 May 19;179(8):886–95. doi: 10.1001/jamapediatrics.2025.1012 (PMC12090068; doi:10.1001/jamapediatrics.2025.1012)
Supplement: Supplement 2. — Data Sharing Statement. [file jamapediatr-e251012-s002.pdf]

Data Sharing Statement

Albaum. Safety Planning Interventions for Suicide Prevention in Children and Adolescents. *JAMA Pediatr*. Published May 19, 2025. doi:10.1001/jamapediatrics.2025.1012

Data
Data available: No

Additional Information
Explanation for why data not available: Available upon reasonable request to the author
